# Cervical Adenoma Malignum in Third Trimester Pregnancy

**DOI:** 10.5334/jbsr.2298

**Published:** 2021-01-06

**Authors:** Katelijn Pannecoeck, Philippe Tummers, Pieter De Visschere

**Affiliations:** 1 UZ Gent, BE

**Keywords:** Gynaecology, Urogenital Radiology, Cervical cancer, Cervical tumor, Cervical adenocarcinoma, Mucinous adenocarcinoma, Pregnancy, Uterine cervix MRI

## Abstract

**Teaching Point:** Adenoma malignum is a rare, non-HPV associated subtype of mucinous adenocarcinoma of the cervix, of which we present an exceptional case that was discovered during the third trimester of pregnancy.

## CASE STUDY

A 35-year-old 38-weeks-pregnant woman was referred to the gynaecology department of our hospital for the diagnostic workout of excessive pre-labor watery vaginal discharge. She had no other clinical symptoms. Lab results were normal. Cervical Papanicolaou cytopathology showed a high number of neutrophils, suggesting an inflammatory etiology, without signs of malignancy. Nevertheless, at clinical examination a posterior cervical mass was palpable.

**Figure 1:**
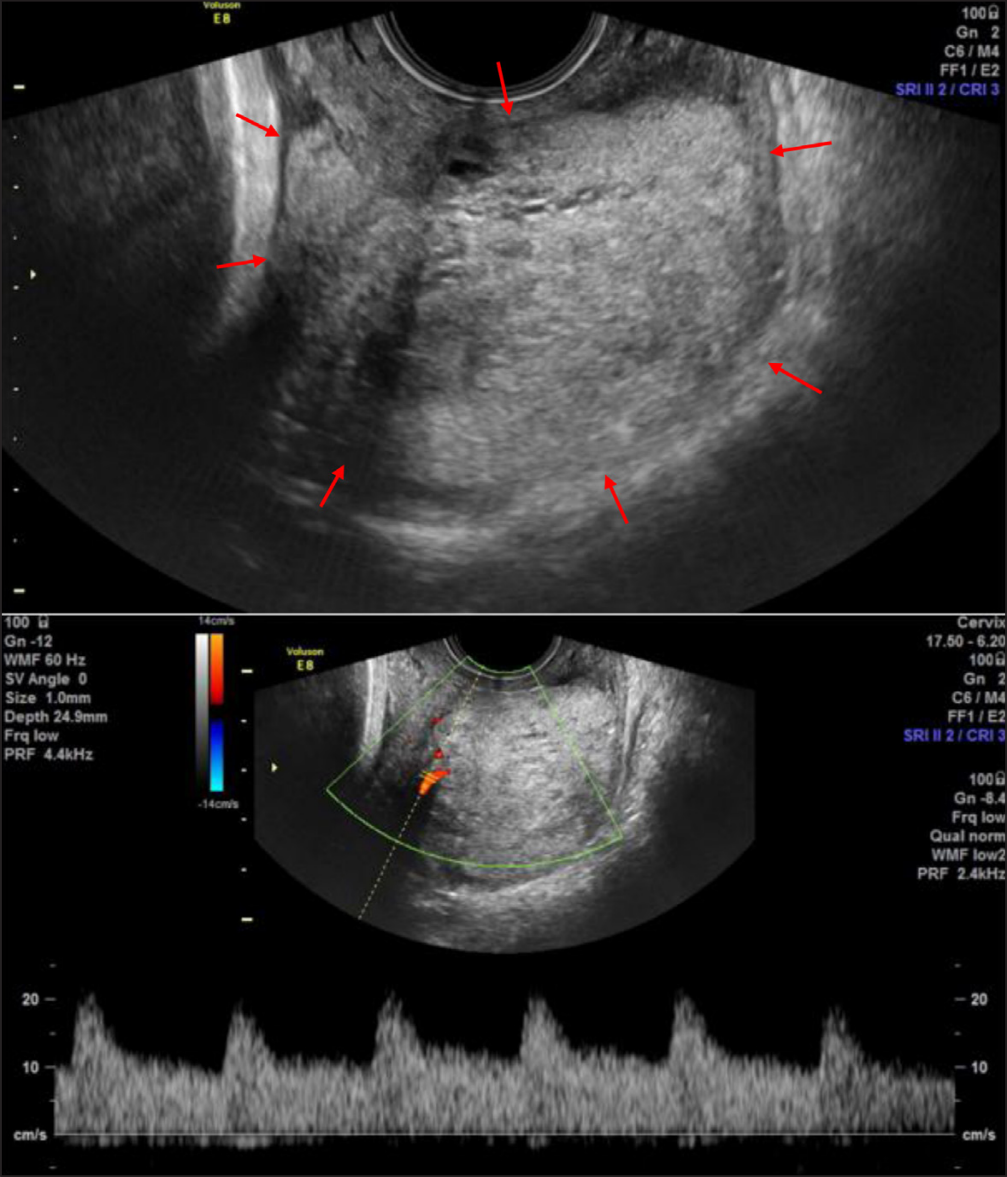


**Figure 2:**
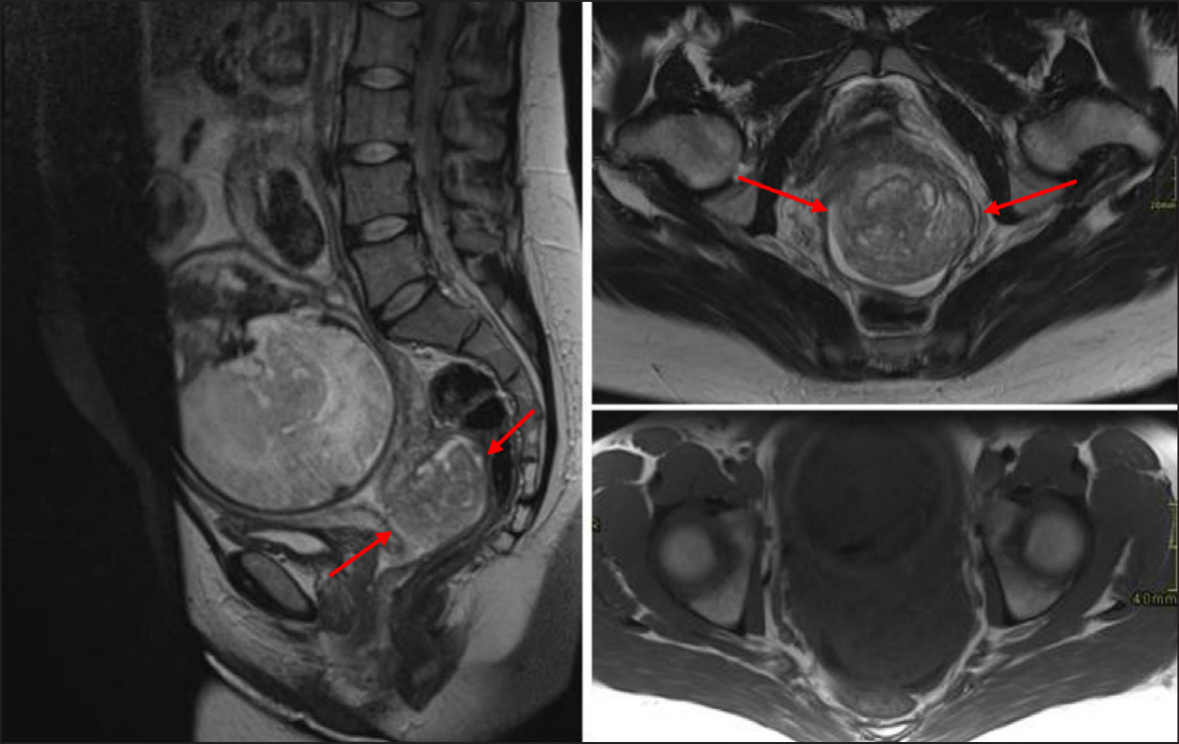


**Figure 3:**
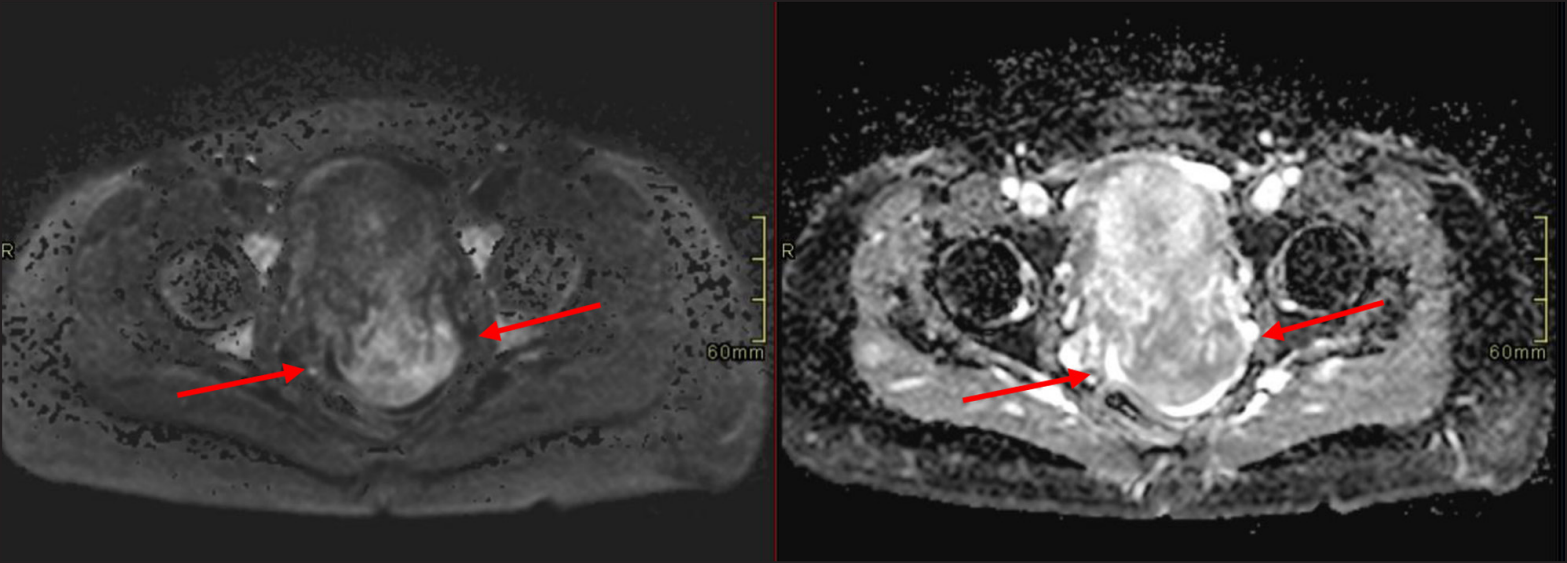


Transvaginal ultrasound confirmed the presence of a large cervical mass (maximum diameter of 5.5 cm) located predominantly on the posterior portion of the cervix. The mass had a slightly heterogenous and hyperechoic appearance, with some small cysts. Color Doppler showed some vascularity (***Figure 1***). Because of the lesion’s size and the upcoming partus, additional magnetic resonance imaging (MRI) was urgently performed. It showed a well-circumscribed mass in the posterior cervical wall (***Figure 2***). The mass was iso-intense to the uterus on T1-weighted imaging. It had a heterogenous signal intensity on T2-weighted images, with multiple T2 hyperintense foci, correlating with the cystic/mucinous lesions seen on ultrasound. A high amount of fluid was demonstrated in the superior portion of the vagina and there was a disruption of the posterior portion of the hypo-intense fibrous cervical stroma, with the mass growing into the posterior fornix of the vagina. There was no invasion of the posterior vaginal wall, the parametria or peritoneum. The mass showed slight diffusion restriction (***Figure 3***). A tru-cut biopsy of the cervical mass was performed and showed a mucinous endocervical adenocarcinoma of the minimal deviation adenocarcinoma subtype, also called adenoma malignum. After delivery (with c-section), hysterectomy, salpingo-ovariectomy and lymphadenectomy were performed.

## COMMENT

The majority of cervical tumors (75–80%) are squamous cell carcinoma, the other 20–25% are adenocarcinomas. Adenoma malignum is a rare, non-HPV (Human Papilloma Virus) associated subtype of mucinous adenocarcinoma of the cervix, arising from the mucin-producing columnar epithelium of the endocervical glands, accounting for 3% of cervical adenocarcinomas [1].

An important clinical finding associated with adenoma malignum, is an excessive amount of watery discharge. The lesion is often associated with mucinous tumors of the ovary and with Peutz-Jeghers syndrome, which is characterized by hyperpigmented macules on the lips and oral mucosa and benign hamartomatous polyps of the gastro-intestinal tract.

Because of the deceptively benign appearance both on imaging (cystic components, no marked diffusion restriction, well circumscribed) and on histopathology, adenoma malignum may be wrongly interpreted as a benign lesion. However, it disseminates early into the peritoneal cavity and more distal sites and has only a minor response to chemotherapy and radiation therapy. The prognosis is thus poor.
